# The draft genome of *Nitzschia closterium* f. minutissima and transcriptome analysis reveals novel insights into diatom biosilicification

**DOI:** 10.1186/s12864-024-10479-9

**Published:** 2024-06-05

**Authors:** Yajun Li, Jinman He, Xiuxia Zhang, Xiaodong Deng

**Affiliations:** 1 Institute of Tropical Bioscience and Biotechnology, Chinese Academy of Tropical Agricultural Sciences (CATAS) & Key Laboratory of Biology and Genetic Resources of Tropical Crops of Hainan Province, Haikou, 571101 China; 2 Hainan Provincial Key Laboratory for Functional Components Research and Utilization of Marine Bioresources, Haikou, 571101 China

**Keywords:** Biosilicification, Draft genome, Frustule, *Nitzschia closterium* f. minutissima, Transcriptome analysis

## Abstract

**Background:**

*Nitzschia closterium* f. minutissima is a commonly available diatom that plays important roles in marine aquaculture. It was originally classified as Nitzschia (Bacillariaceae, Bacillariophyta) but is currently regarded as a heterotypic synonym of *Phaeodactylum tricornutum*. The aim of this study was to obtain the draft genome of the marine microalga *N. closterium* f. minutissima to understand its phylogenetic placement and evolutionary specialization. Given that the ornate hierarchical silicified cell walls (frustules) of diatoms have immense applications in nanotechnology for biomedical fields, biosensors and optoelectric devices, transcriptomic data were generated by using reference genome-based read mapping to identify significantly differentially expressed genes and elucidate the molecular processes involved in diatom biosilicification.

**Results:**

In this study, we generated 13.81 Gb of pass reads from the PromethION sequencer. The draft genome of *N. closterium* f. minutissima has a total length of 29.28 Mb, and contains 28 contigs with an N50 value of 1.23 Mb. The GC content was 48.55%, and approximately 18.36% of the genome assembly contained repeat sequences. Gene annotation revealed 9,132 protein-coding genes. The results of comparative genomic analysis showed that *N. closterium* f. minutissima was clustered as a sister lineage of *Phaeodactylum tricornutum* and the divergence time between them was estimated to be approximately 17.2 million years ago (Mya). CAFF analysis demonstrated that 220 gene families that significantly changed were unique to *N. closterium* f. minutissima and that 154 were specific to *P. tricornutum*, moreover, only 26 gene families overlapped between these two species. A total of 818 DEGs in response to silicon were identified in *N. closterium* f. minutissima through RNA sequencing, these genes are involved in various molecular processes such as transcription regulator activity. Several genes encoding proteins, including silicon transporters, heat shock factors, methyltransferases, ankyrin repeat domains, cGMP-mediated signaling pathways-related proteins, cytoskeleton-associated proteins, polyamines, glycoproteins and saturated fatty acids may contribute to the formation of frustules in *N. closterium* f. minutissima.

**Conclusions:**

Here, we described a draft genome of *N. closterium* f. minutissima and compared it with those of eight other diatoms, which provided new insight into its evolutionary features. Transcriptome analysis to identify DEGs in response to silicon will help to elucidate the underlying molecular mechanism of diatom biosilicification in *N. closterium* f. minutissima.

**Supplementary Information:**

The online version contains supplementary material available at 10.1186/s12864-024-10479-9.

## Background

Diatoms (Bacillariophyceae) are photosynthetic unicellular eukaryotes, that constitute a dominant group of marine phytoplankton. They play an extremely important role in the matter cycle and energy flow of ecosystems. It is estimated that diatoms contribute approximately 20% of total primary production and as much as 40% of particulate organic carbon export [1]. In addition, the measurement of global silica production is mostly supported by diatoms, which consume silicic acid to precipitate biogenic silica as their siliceous cell wall [2]. *Nitzschia closterium* f. minutissima is a common coastal diatom species that is widely used to feed bivalves, shellfish and copepods in aquaculture hatcheries because of its small size (∼15 μm), rapid growth rate, high oil content and excellent environmental adaptability [3, 4]. It was originally classed as Bacillariophyta/Bacillariophyceae/Bacillariales/Bacillariaceae/Nitzschia but has currently been suggested to be a strain of *Phaeodactylum tricornutum* (Phaeodactylaceae, Bacillariophyceae ordo incertae sedis) [5]. *P. tricornutum* has become a model organism for diatom molecular studies with well-characterized genomic, metabolic and cellular features [6]. In contrast, little information is available about *N. closterium* f. minutissima, and deciphering its genome is a crucial step toward better understanding its evolution and biology.

With the development of high-throughput sequencing technologies, numerous genomic sequencing projects of diatoms have been performed to illustrate genome variation, evolution and adaptation, accompanied by sets of transcriptomic data obtained under various growth conditions. To data, a total of 69 diatom genome sequences have become available in NCBI databases. Based on these genomes, transcriptomic analyses have been extensively used to explore molecular mechanisms underlying various biological questions such as nutrient starvation (N, P, Fe, Cu, Si), abiotic stress (elevated CO_2_ concentration, pH change, salinity stress, low/or high temperature and light exposure), sexual reproduction and distinct developmental stages in diatoms [7–10]. In recent years, scientists have been intrigued to determine the detailed biological processes and molecular mechanisms involved in the formations of cell wall (also called frustule) in diatoms since frustules are considered promising next-generation nanoscale materials for a variety of applications, ranging from drug delivery to bone repair, biosensors, metal nanoparticles and optoelectric devices [11–15]. Diatom frustules comprise two valves closely joined together by girdle bands that typically surround the cell, exhibiting intricate, ornate and species-specific features at the micro- and nanometer scales [16], whose formations have been demonstrated to be under strict biological control [17].

*Thalassiosira pseudonana*, whose genome was first sequenced from diatoms [18], was the most advanced model species for the studies of these biosilicification processes. In general, these processes occur inside silica deposition vesicles (SDVs) which are intercellular membrane-bound. Upon maturation, the new valve or girdle band is exocytosed and assembled into a cell wall [19, 20]. However, the formation of *Chaetoceros tenuissimus* setae is not mediated by an SDV, implying that a different extracellular silicification mechanism exists in diatom species [21]. Transcriptomic and proteomic analyses revealed that numerous genes and proteins participate in the morphogenesis of frustules, and several related proteins, such as silaffins, ankyrin repeat domain proteins (dAnks) and silicalemma-associated proteins (SAPs), were identified. Silaffin-1 is a highly conserved protein of the SDV membrane and contributes to the strength and stiffness of frustules [22]. However, dAnks were predicted to bind to the cytosolic domain of a transmembrane protein and be responsible for the biosynthesis of pore patterns in diatom biosilica [23], and the SAP1 and 3 knockdown lines presented malformed valves in *T. pseudonana* [24]. In addition, proteins including cingulins, long-chain polyamines (LCPAs), silacidins, and the cytoskeleton, were also proven to play critical roles in the formation of frustules [25–28]. Notably, Skeffington et al. (2022) [29] reported that the amino acid sequences of isolated silica-associated proteins exhibited low similarity among diatom species but shared unconventional sequence motifs that may have similar functions. Moreover, posttranscriptional regulation is also involved in silicon biomineralization since many genes were found to exert their functions via alternative polyadenylation [30]. However, despite significant progress in recent years, the molecular basis underlying biosilicification in diatoms is still largely uncharacterized.

In the present study, to extend the knowledge about the molecular processes involved in biosilicification, a draft genome sequence was obtained, and transcriptome analysis of the silicon responses of *Nitzschia closterium* f. minutissima was performed. Comparative genomic analysis, including the construction of a phylogenetic tree, estimation of divergence times and analysis of expanded and contracted gene families, was carried out to investigate the phylogenetic placement of *N. closterium* f. minutissima, and determine the evolutionary specialization between *N. closterium* f. minutissima and *P. tricornutum*. Then, RNA sequencing (RNA-seq) was performed to identify differentially expressed genes (DEGs) during biosilicification in diatoms. Numerous genes involved in various biological processes, such as transcriptional regulation and glycolysis, were up-or down regulated after 6 h and 12 h of cultivation in silicon replenishment media, providing novel insights into diatom biosilicification.

## Results

### Genome sequencing, assembly and annotation of *N. closterium* f. minutissima

A total of 13.81 Gb of pass reads were generated from the PromethION sequencer, covering approximately 157.9 × of the genome (Table 1). The genome size of *N. closterium* f. minutissima was estimated to be 29.28 Mb for 27 contigs, with an N50 contig length of 1.23 Mb. In addition, the GC content of the genome assembly was 48.55% (Table 1). Overall, 5.37 Mb of repetitive sequences representing 18.36% of the genome were identified, of which the most abundant were long terminal repeats (LTRs) (14.36%), followed by DNA transposons (2.34%) and unknown classified repeats (0.66%) (Table S1).

A total of 9,132 genes were predicted from the repeat-masked genome, and 6,738 genes were functionally annotated using five public databases, namely, the Swissprot, Kyoto Encyclopedia of Genes and Genomes (KEGG), Eukaryotic Orthologous Group of Protein (KOG), Gene Ontology (GO) and Nonreduntant Protein (NR) Databases. The average gene length of the predicted genes was 1,674, the average CDS length was 1,556, the average number of exons per gene was 1.65, and the average exon length was 941(Table 1). In addition, 142 noncoding RNAs (ncRNAs) were annotated, including 92 transfer RNAs (tRNAs), 28 ribosomal RNAs (rRNAs), 10 small RNAs and 12 regulatory elements (Table 1). The genome assembly has been deposited in GenBank with the accession number JARGZD000000000 under the BioProject PRJNA943072. The taxon name *N. closterium* f. minutissima was revised to *Phaeodactylum tricornutum* in the NCBI database because it is currently regarded as a heterotypic synonym of *P. tricornutum* [5]. Hence, *Phaeodactylum tricornutum_*PRJNA943072 is used hereinafter instead of *N. closterium* f. minutissima.

**Table 1:** Summary statistics of the Assembly and Annotation of *Nitzschia closterium* f. minutissima genome

Assembly	
Total pass reads (Gb)	13.81
Coverage depth	157.9
Total assembly size(Mb)	29.28
Contigs number	28
Contigs N50 (Mb)	1.23
GC (%)	48.55%
Annotation	
Predicted genes number	9,132
Annotated genes number	6,738
Average gene length (bp)	1,674
Average CDS length (bp)	1,556
Average exons number per gene	1.65
Average exon length (bp)	942
tRNA	92
rRNA	27
Small RNA	10
Regulatory	12

### Comparative genomic analysis between nine diatom species

**Fig. 1:**
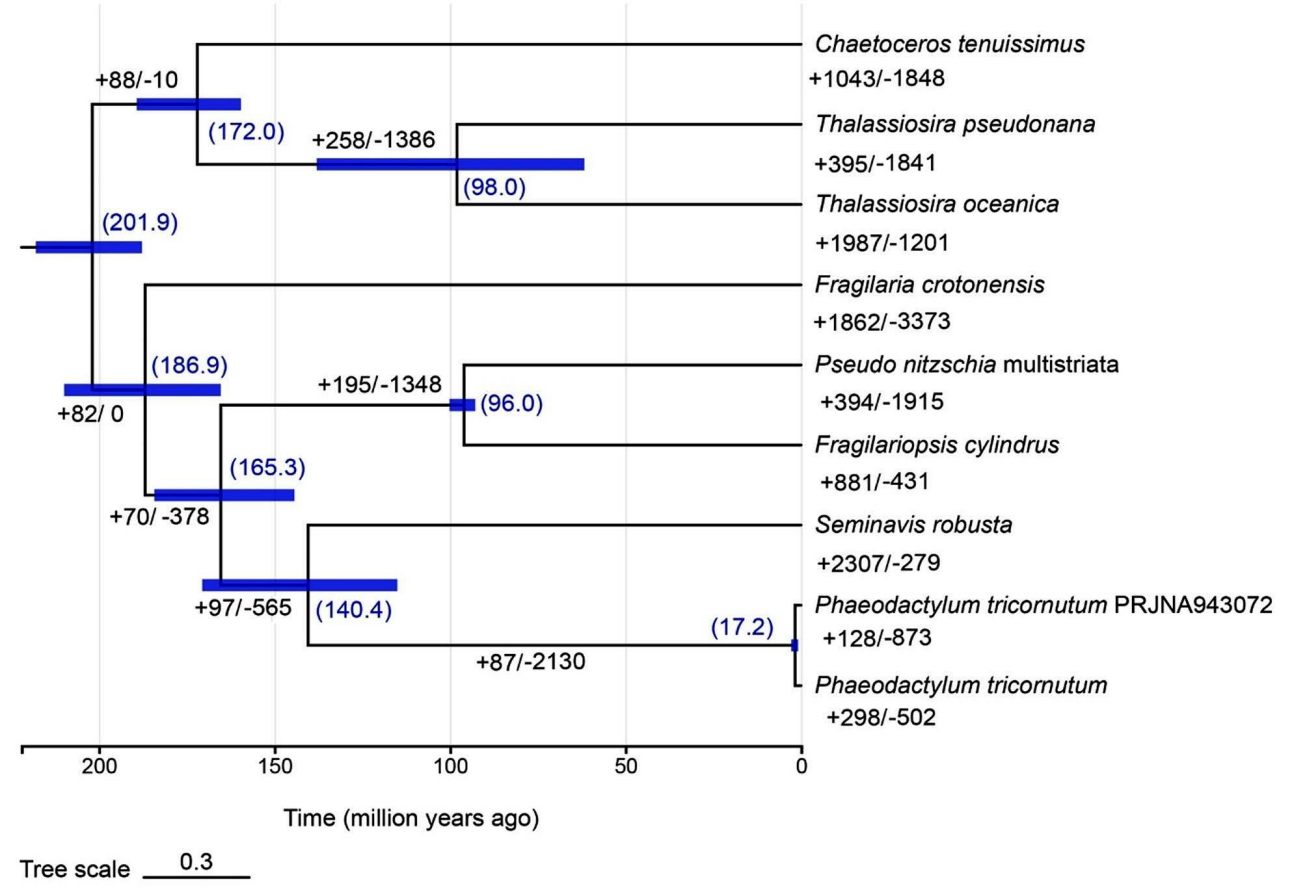
Phylogenetic tree of *Phaeodactylum tricornutum*_PRJNA943072 and other 8 diatom species based on the 926 single-copy orthogroups. The estimated divergence time (million years ago, Mya) is shown as the blue numbers in the brackets and plotted at each node. The blue bars represent the 95% confidence interval of divergence time. Expansion and contraction of gene family are denoted as numbers with plus and minus signs (+ and -), respectively

To investigate the phylogenetic placement of *P. tricornutum*_PRJNA943072 among diatom species, a phylogenetic tree was constructed on the basis of 926 single-copy orthologues identified from *P. tricornutum*_PRJNA943072 and 8 other diatom species whose whole-genome protein sequences are available in the NCBI database, where *P. tricornutum*_PRJNA943072 was clustered as a sister lineage of *P. tricornutum* and subsequently formed a clade with *Seminavis robusta* belonging to the order Naviculales (Fig. 1, Table S2). These genes were closely related to two Bacillariales species, *Fragilariopsis cylindrus* and *Pseudo nitzschia multistriata*. These three species belong to the class Bacillariophyceae and were clustered with another Bacillariophyceae species, *Fragilaria crotonensis*, separated from a clade consisting of three class Mediophyceae species, *T. oceanica*, *T. pseudonana* and *C. tenuissimus* (Fig. 1, Table S2). The divergence times of these diatoms were further estimated using MCMCTREE in PAML v4.9j. The results showed that the divergence time between *P. tricornutum*_PRJNA943072 and *P. tricornutum* was estimated to be approximately 17.2 million years ago (Mya), which further diverged from the common ancestor with *S. robusta* at 140.4 Mya (Fig. 1).

**Fig. 2:**
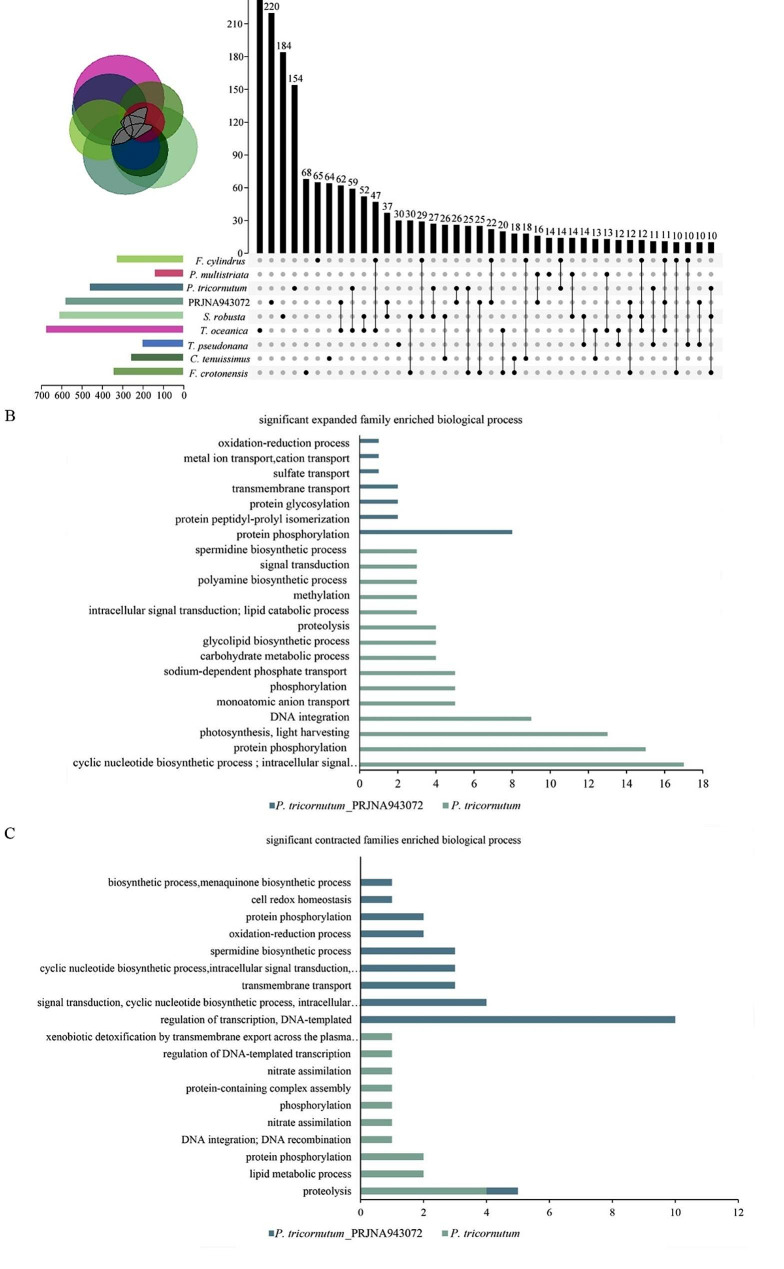
An analysis of significant expansion and contraction of gene families (*P* < 0.05). (**A**) Venn and upset plot diagrams of significant expansion and contraction of gene families among nine diatom species. The min overlap set size was 10. (**B**) GO enrichment analysis for biological process of significant expanded gene families specific to *P. tricornutum*_PRJNA943072 and *P. tricornutum* (Top 22). (**C**) GO enrichment analysis of significant contraction gene families specific to *P. tricornutum*_PRJNA943072 and *P. tricornutum* (Top 20)

To illustrate the evolutionary specialization, expansion and contraction of gene families were analyzed using CAFE v5.0 among nine diatom species including *P. tricornutum*_PRJNA943072. A total of 21,437 orthogroups containing 155,880 genes were identified across these diatom species, among which 2,951 orthogroups were present in all species and 6,888 were species specific. In the genome of *P. tricornutum*_PRJNA943072, 128 gene families underwent expansion, and 873 gene families underwent contraction. Howerer, expansions of 298 gene families and contractions of 502 gene families were detected in *P. tricornutum* (Fig. 1), suggesting differences in the genomes of these two species. To further reveal the differences in gene families, significantly expanded and contracted gene families were extracted (*P* < 0.05), and GO enrichment was carried out for biological processes in the genomes of *P. tricornutum*_PRJNA943072 and *P. tricornutum*. The results showed that 220 gene families that significantly changed were unique to *P. tricornutum*_PRJNA943072 and that 154 were specific to *P. tricornutum*. Only 26 gene families overlapped between these two species (Fig. 2A). GO enrichment demonstrated that the significantly expanded gene families were related to protein phosphorylation (GO:0006468), protein peptidyl-prolyl isomerization (GO:0000413), protein glycosylation (GO:0006486), transmembrane transport (GO:0055085), sulfate transport (GO:0008272)/metal ion transport (GO:0030001), cation transport (GO:0006812) and oxidation-reduction processes (GO:0055114) in *P. tricornutum*_PRJNA943072, and the top 3 GO terms were cyclic nucleotide biosynthetic process (GO:0009190)/intracellular signal transduction (GO:0035556), protein phosphorylation (GO:0006468) and photosynthesis/light harvesting (GO:0009765) in *P. tricornutum* (Fig. 2B). The significantly contracted gene families were enriched in GO terms such as regulation of transcription/DNA-templated (GO:0055114), signal transduction/cyclic nucleotide biosynthetic process/intracellular signal transduction (GO:0055085) and transmembrane transport (GO:0045454) in *P. tricornutum*_PRJNA943072, whereas proteolysis (GO:0006508), lipid metabolic process (GO:0015074 and GO:0006310) and protein phosphorylation (GO:0006629) were enriched in *P. tricornutum* (Fig. 2C).

### Transcriptome analysis

**Fig. 3:**
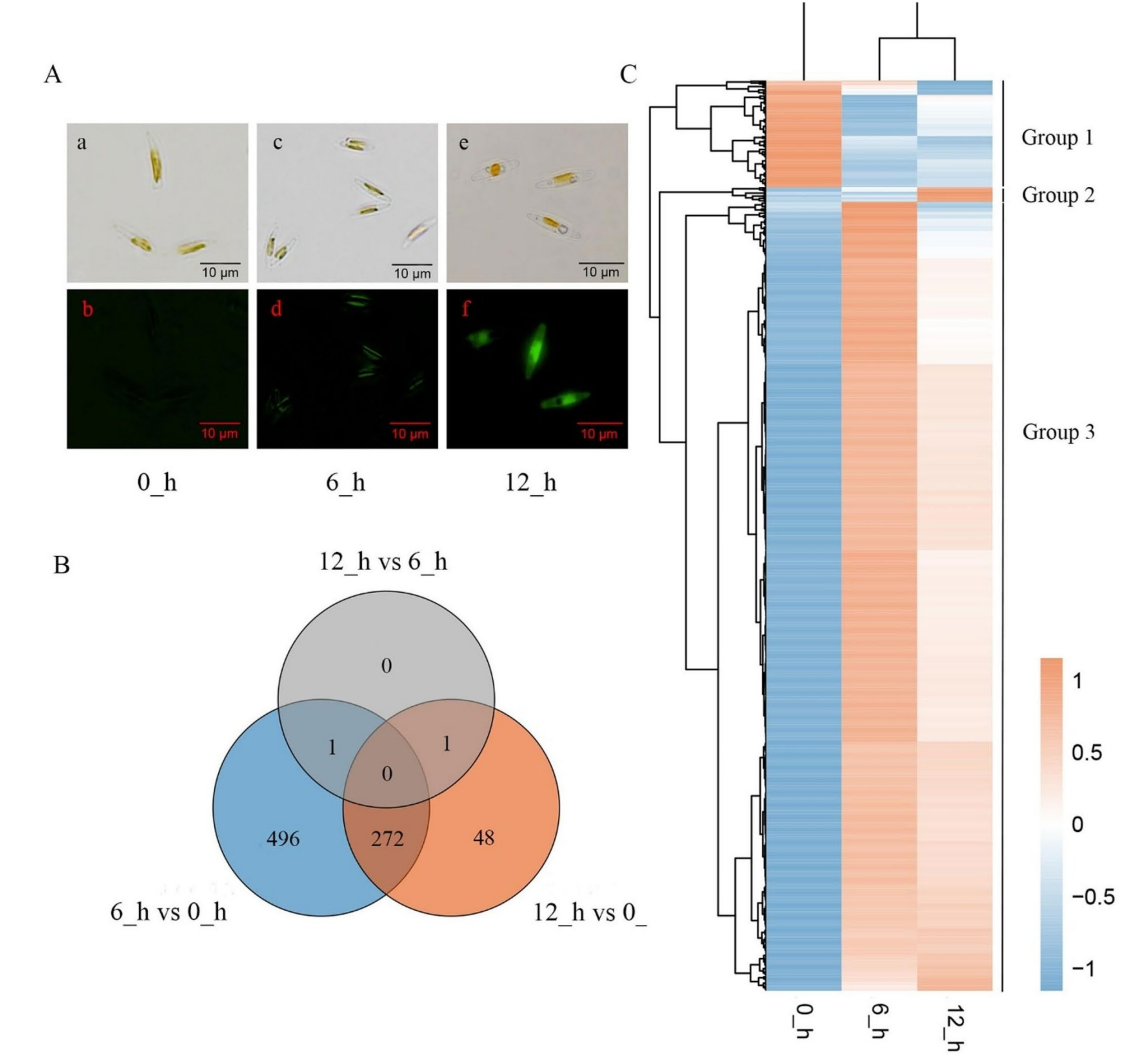
Microscopic and transcriptome analysis of synchronized cells of *N. closterium* f. minutissima after 0, 6 and 12 h of cultivation in 1/2f medium under normal silicate condition. Cells were stained by rhodamine 123 and microscopic images (**A**) were captured at 0, 6 and 12 h timepoints, bar = 10 μm; (a), (c) and (e), images obtained under bright field; (b), (d) and (f), images obtained under dark field. The new cell wall was detected by green fluorescence. Venn diagram (**B**) and heatmap (**C**) of significantly differentially expressed genes extracted from RNA-seq data. The data from 0_h was used as the control. Corrected P-value of 0.05 and absolute foldchange of 2 were set as the threshold for significantly differential expression and the experiment was repeated three times

In this study, a silicon starvation-replenishment procedure was used for cell synchronization as previously described for *T. pseudonana* [31]. To monitor new cell wall formation in synchronized cells, fluorescence images of cells stained with rhodamine 123 were captured via fluorescence microscopy. As shown in Fig. 3A, no fluorescence was observed in the cells cultured for 0 h, suggesting that silicon starvation led to cell cycle arrest in *P. tricornutum*_PRJNA943072. As the culture time increased to 6 h after silicon was added back to the medium, the algal cells exhibited green fluorescence, revealing that they resumed their growth upon silicon replenishment. When the synchronized cells were maintained in silicon-containing medium for 12 h, they emitted strong green fluorescence throughout the whole cells, implying that a new cell wall had formed.

Subsequently, transcriptome analysis through RNA-seq was performed to elucidate the genes whose expression significantly differed during cell wall silicification. Synchronized algal cells were sampled after 0, 6 and 12 h of cultivation under silicon replenishment conditions. The data derived from 0 h was used as a control. A total of 818 genes were significantly up- or downregulated and 272 genes were shared after 6 h and 12 h. A total of 496 genes exhibited specific expression changes after 6 h and 48 genes exhibited changes after 12 h (Fig. 3B, Table S3). The number of genes in the former was more than ten times of that in latter. Notably, only two genes were significantly enriched after 12 h, compared with those after 6 h (Fig. 3B). The results suggested that a majority of the genes were activated in response to silicon before 6 h. According to the gene expression profiles, these genes were divided into three groups: Group 1 was downregulated after 6 and 12 h; Group 2 was induced or upregulated after 12 h; and Group 3 was specifically induced or upregulated after 6 h (Fig. 3C). The majority of the identified genes were assigned to Group 3, revealing that the responses to silicon were positively triggered in diatom cells, to some extent.

### GO enrichment and KEGG pathway analysis of the significantly differentially expressed genes

**Fig. 4:**
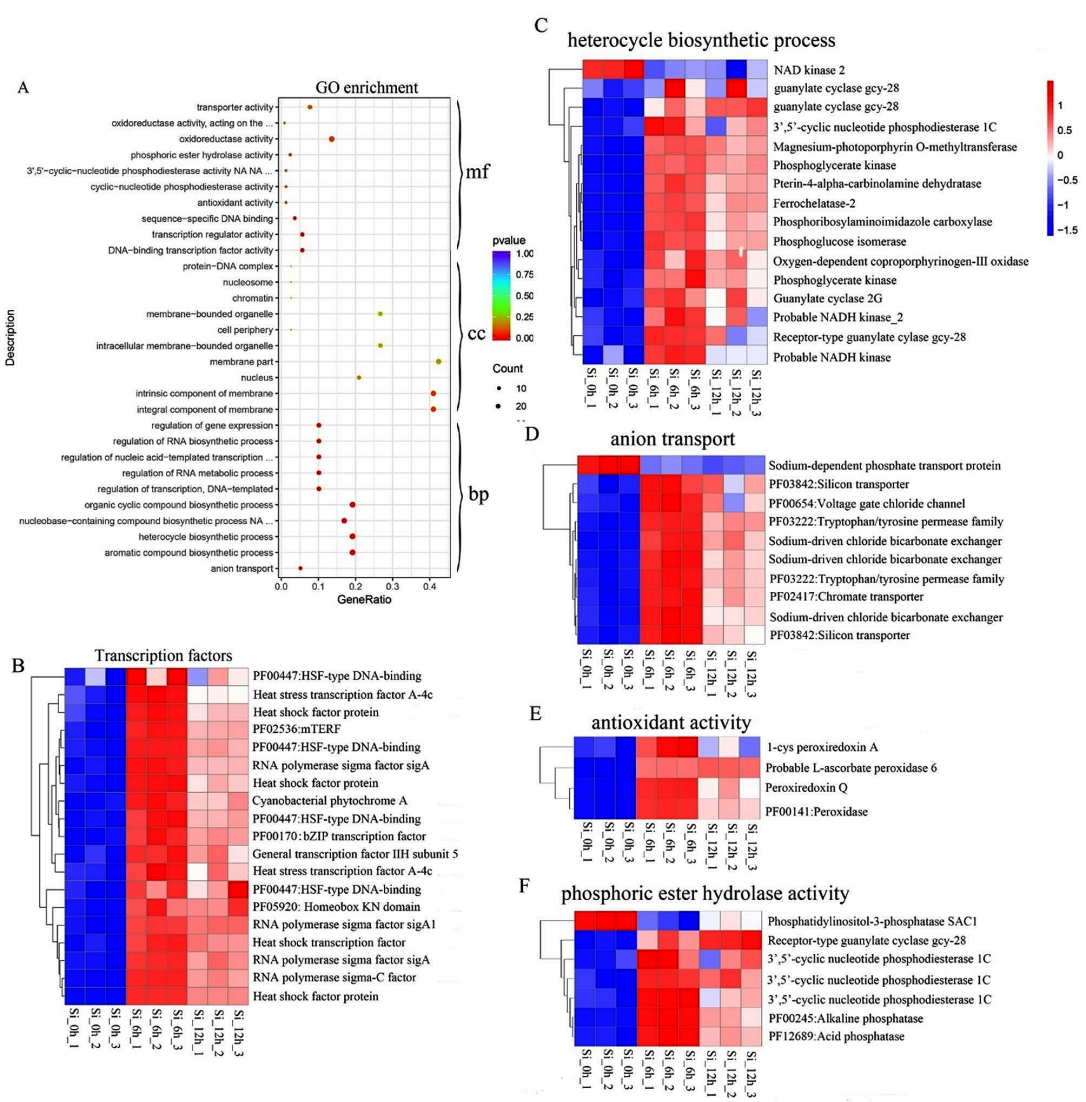
GO enrichment (**A**) and heatmap of the corresponding genes that significantly enriched in biological process and molecular function (**B**-**F**). GO category: mf, molecular function; cc, cellular component; bp, biological process. Top 10 of GO term in every category were shown. Si_0h_1, Si_0h_2 and Si_0h_3 represented three repeated samples that were harvested from 24 h silicon-starvation synchronized cultures; Si_6h_1, Si_6h_2 and Si_6h_3 represented three repeated samples that were maintained for 6 h in silicon replenishment media after 24 h silicon-starvation synchronization. Si_12h_1, Si_12h_2 and Si_12h_3 represented three repeated samples that were maintained for 12 h after synchronization

**Fig. 5:**
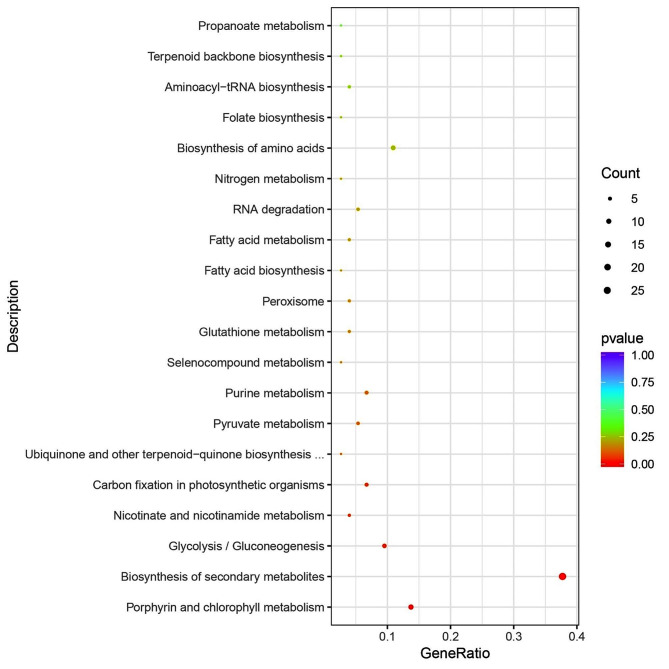
KEGG pathway enrichment analysis of significantly differentially expressed genes (Top 20)

GO enrichment and KEGG pathway analyses were subsequently performed to further understand the functions of the genes whose expression was significantly altered in response to silicon. The results of GO enrichment showed that genes associated with the following GO terms were overrepresented in the biological process category: “regulation of gene expression”, “regulation of RNA biosynthetic process”, “regulation of nucleic acid-templated transcription”, “regulation of RNA metabolic process” and “regulation of transcription, DNA-templated”. The corresponding enrichment was detected in the molecular function category, which included “sequence-specific DNA binding”, “transcription regulator activity” and “DNA-binding transcription factor activity” (Fig. 4A, Table S4). The results suggested that genes encoding transcription factors were significantly enriched during biosilicification in *P. tricornutum*_PRJNA943072. Interestingly, about approximately half of the genes were heat shock factor genes, all of which were upregulated after 6 h and 12 h of cultivation in silicon-containing media (Fig. 4B). In the biological process category, genes involved in “organic cyclic compound biosynthesis process”, “nucleobase-containing compound biosynthetic process”, “heterocycle biosynthetic process” and aromatic compound biosynthetic process” were also overrepresented. Moreover, these genes encoded the transcription factors mentioned above (Fig. 4A, Table S4). In addition, genes encoding proteins, including guanylate cyclase, glycolysis and photosynthesis related proteins such as phosphoglycerate kinase and magnesium-protoporphyrin O-methyltransferase, were significantly enriched. In addition, only NAD kinase 2 was downregulated after 6 h and 12 h of cultivation and the other genes were upregulated (Fig. 4C). Obvious enrichment was also observed for anion transport in the biological process category, among which four genes were related to chloride transport and exchange, two genes encoded silicon transporters (SITs) and one gene encoded a sodium-dependent phosphate transport protein that was downregulated after 6 h and 12 h (Fig. 4D). The results suggested that silicon transport was accompanied by the activation of chloride channels and chloride/bicarbonate exchange, as well as the suppression of the Na+/Pi cotransporter. In the molecular function category, genes encoding proteins involved in antioxidant activity (Fig. 4E) and phosphoric ester hydrolase activity (Fig. 4F) were significantly enriched, and included various peroxiredoxins, phosphodiesterases and phosphatases, implying that oxidation-reduction reactions and dephosphorylation were activated during diatom biosilicification. According to the KEGG enrichment analysis, five pathways were significantly enriched: Porphyrin and chlorophyll metabolism, Biosynthesis of secondary metabolites, Glycolysis/Gluconeogenesis, Nicotinate and nicotinamide metabolism and Carbon fixation in photosynthetic organisms (Fig. 5).

### Genes related to cytoskeleton-associated proteins, epigenetic modification, protein interaction, carbohydrate metabolism and fatty acid metabolism and desaturase

**Fig. 6:**
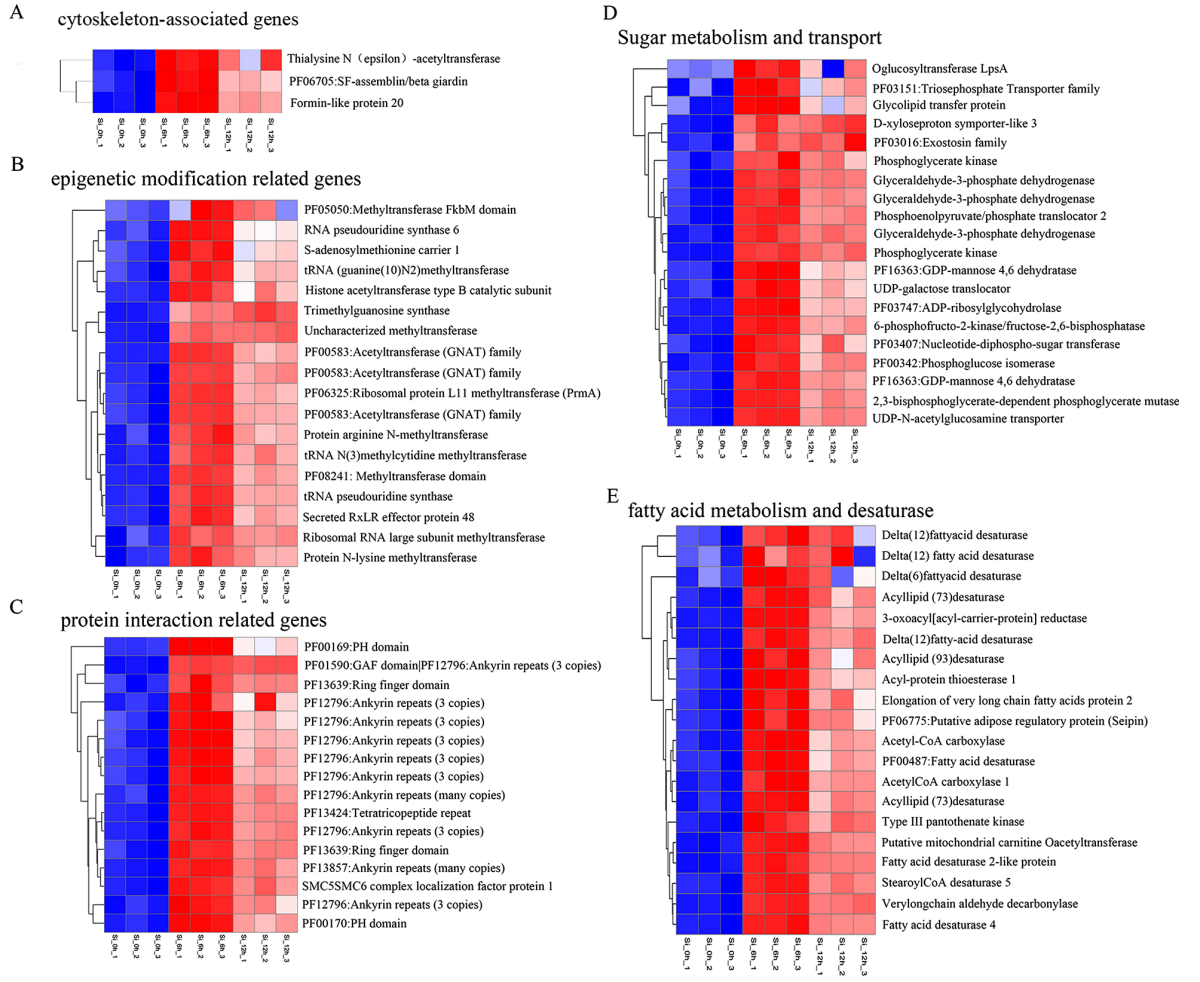
Heatmap of genes encoding proteins involved in cytoskeleton-associated genes (**A**), epigenetic modification (**B**), protein interaction (**C**), sugar metabolism and transport (**D**), and fatty acid metabolism and desaturase (**E**)

To further analyze the transcriptional changes that occur during diatom biosilicification, genes involved in cytoskeleton-associated proteins, epigenetic modifications, protein interactions, sugar metabolism and transport, and fatty acid metabolism and desaturase were identified (Fig. 6). Three genes encoding proteins including thialysine N (epsilon)-acetyltransferase which catalyzes the acetylation of polyamines [32], SF-assemblin/beta giardin and formin-like protein 20, which are cytoskeleton-associated proteins [33, 34] were up regulated after 6 h and 12 h (Fig. 6A). These results implied that ployamines and cytoskeleton components such as microtubule and microfilament would play important roles in cell wall formation in diatoms. Several methyltransferase and acetyltransferase genes, such.

as PF05050: Methyltransferase FkbM domain and Histone acetyltransferase type B, were also upregulated after 6 h (Fig. 6B), suggesting that epigenetic modification likely positively contributed to the process of biosilicification in diatoms [35]. Notably, ten genes encoding proteins containing an ankyrin repeat domain that mainly mediates protein interactions [36] were upregulated (Fig. 6C). For sugar metabolism and transport, approximately half of the genes, such as those encoding glyceraldehyde-3-phosphate dehydrogenase and phosphoglycerate kinase, are involved in glycolysis [37]. In addition, the GDP-mannose 4,6 dehydratase, UDP-N-acetylglucosamine transporter and UDP-galactose translocator genes were upregulated after 6 h and 12 h (Fig. 6D), reflecting that glycolysis, fucose biosynthesis and D-xyloseproton and galactose transport were triggered during diatom biosilicification [38, 39]. On the other hand, the transcript levels of several fatty acid biosynthesis and desaturase genes increased after silicon replenishment (Fig. 6E). These results indicated that carbon flow dramatically changed after silicon replenishment in *P. tricornutum*_PRJNA943072.

## Discussion

*N. closterium* f. minutissima is a marine economic microalgae and plays important roles in marine aquaculture. It was originally assigned to the genus Nitzschia (Bacillariales, Bacillariophyceae), but recently changed to a heterotypic synonym of *P. tricornutum* since DNA barcode sequences such as 18 S rDNA, actin genes and internal transcribed spacer (ITS) sequences derived from *N. closterium* f. minutissima shared greater similarities with those of *P. tricornutum* than Nitzschia species [5]. In the present study, draft genome sequences of *N. closterium* f. minutissima were obtained, and the size was 29.28 Mb (Table 1), which was slightly larger than the 27.5 Mb length of *P. tricornutum* [40]. Moreover, the phylogenetic analysis revealed that *N. closterium* f. minutissima (*P. tricornutum*_PRJNA943072) was more closely related to *P. tricornutum*, and these two lineages have only diverged for 17.2 million years (Fig. 1), consistent with previous findings. However, it is estimated that new diatom species can evolve within as little as 4000 years [41], therefore, it was difficult to assume that *N. closterium* f. minutissima was the same species as *P. tricornutum* in this study. Moreover, these two genomes exhibited significant differences in the expansion and contraction of gene families (Fig. 2), implying that they underwent unique evolutionary pressure. It is also likely that some of these differences would be caused by different genome assembly technologies used for *N. closterium* f. minutissima and *P. tricornutum*, and more precise information would be provided with the development of high throughput sequencing and genome assembly technology in future.

To date, a sophisticated procedure for cell synchronization has been constructed for *T. pseudonana*, which was used as a model organism for the study of biosilicification [31]. In the present study, similar results were obtained for *P. tricornutum*_PRJNA943072 that silicon starvation for 24 h arrested cell cycle progression, and the cells continued growing upon silicon replenishment (Fig. 3A). According to RNA-seq results, cells exposed to silicon seemed to undergo early triggering since most of the DEGs were induced or upregulated after 6 h of cultivation when silicate was present, and fewer genes were activated as the cultivation time increased to 12 h (Fig. 3B and C). In the case of *T. pseudonana*, girdle band formation continued until approximately 3 h, after which the cells began to synthesize at 4 h, and the cells separated approximately 7 h after the silicon was added. In *P. tricornutum*_PRJNA943072, 6 h may be an important timepoint for cell wall formation.

GO enrichment analysis revealed that several transcription factors, especially heat shock factors (HSFs), were significantly enriched, followed by genes involved in the biological processes of heterocycle biosynthetic process and anion transport (Fig. 4A and D). HSF is an important gene family that plays crucial roles in plant responses to various stresses, as well as plant growth and development. The expression of these genes has been reported to be significantly upregulated by the exogenous application of silicon to higher plants, such as tomato and date palm, in response to heat stress, where it contributes to preventing excessive reactive oxygen species (ROS) accumulation and membrane lipid peroxidation [42]. Remarkably, heat shock proteins might affect membrane fluidity to modulate the properties of the SDV membrane [23]. On the other hand, two silicon transporters (SIT) genes were identified and upregulated after silicate was added (Fig. 4D). SITs are localized in the plasma membrane underneath silicified frustules and specifically transport monosilicic acid (Si(OH)4) through the lipid bilayer [43]. In addition, chlorine transport coexists with silicon transport in *P. tricornutum*_PRJNA943072 since the expression pattern of the gene involved in voltage-gated chloride channels was similar to that of SIT gene (Fig. 4D). Moreover, the intracellular pH could be maintained during silicic acid transport, likely by inducing the activation of sodium-driven chloride bicarbonate exchanger because these genes were upregulated and had similar expression patterns to that of SIT gene (Fig. 4D). Currently, little information is available on how silicon signals are transmitted into cells. In the present study, several genes encoding proteins related to cGMP formation and degradation, such as guanylate cyclase and calcium/calmodulin-dependent 3’,5’-cyclic nucleotide phosphodiesterase 1 C, were identified (Fig. 4C and F). Thus, we deduced that second messengers, especially cGMP-mediated signaling pathways, are implicated in signal transduction during diatom biosilicification. On the other hand, genes encoding peroxidases that can reduce hydrogen peroxide and other hydroperoxides were also induced. It is presumed that these genes would be employed to activate antioxidant defense systems to buffer the negative effects, such as ROS accumulation induced by silicon starvation, which is used for cell synchronization. KEGG pathway enrichment of genes related to porphyrin and chlorophyll metabolism and carbon fixation in photosynthetic organisms demonstrated that photosynthesis was enhanced after silicon recovery (Fig. 5). This result was consistent with that of silicon promoted light harvesting for photosynthesis in diatoms [29]. However, little direct evidence has been found concerning whether the genes involved in stress defense or photosynthesis are associated with frustule formation. These physiological processes are likely to be induced for normal cell growth upon silicon replenishment, providing the necessary basis for further silicon uptake, transport and biomineralization.

Except for the two SIT genes, the gene encoding the formin-like protein was also upregulated in this study (Fig. 6A). Formins are transmembrane proteins that participate in actin and microtubule organization by anchoring the cortical cytoskeleton across the membrane to the cell wall [34]. Another cytoskeleton component, the SF-assemblin protein, which constitutes striated microtubule-associated fibres in many flagellate algae [33], appears to play a positive role in the non-flagellate *P. tricornutum*_PRJNA943072 (Fig. 6A). In addition, Thialysine N (epsilon)-acetyltransferase is a rate-limiting enzyme in polyamine homoeostasis, and long-chain polyamines are abundant components of diatom frustules [26]. Interestingly, a series of genes encoding methyltransferases involved in epigenetic modification showed significant differential expression in response to silicon (Fig. 6B). This was consistent with the result of Nemoto et al. (2019) [44] that seven diatom-specific methyltransferases genes were identified according to the transcriptome data and they were suggested to regulate the functions of cell wall formation-related proteins and long-chain polyamines. To data, only a few genes have been functionally characterized in diatom biosilicification, including *dAnks*, *silaffins*, *cingulins* and *silicalemma-associated* (*SAPs*) [22–25]. Among of them, three dAnk proteins controlled the pore patterns in *T. pseudonana* frustule [23]. In the present study, ten *dAnks* gene were significant differentially expressed (Fig. 6C), implying that *P. tricornutum*_PRJNA943072 likely followed similar strategies for the pore patterns biosynthesis. The further functional studies of these ten dANKs genes would provide novel insight for understanding the underlying molecular mechanisms. The other functionally defined genes were not identified in our study. The possible reason is that they might have no changes at transcriptional levels at 6 h after silicon added back.

Apart from these, two series of genes have attracted considerable interest among those significantly upregulated genes. One is group participates in the metabolism and transport of sugars, which include xylose, fucose, galactose and the intermediate product of glycosis. The other is related to fatty acid metabolism and desaturase, especially containing a subset of fatty acid desaturases (Fig. 6D and E). Several studies have demonstrated that glycoproteins and fatty acids are embedded in or closely associated with frustules during the precipitation of silica in diatoms [45–48]. The sugars derived from the diatom frustules were composed of more than 10 polysaccharides, such as xylose, mannan, galactose, rhamnose and fucose, and mannans were considered as the conserved components. The frustule-associated lipids had similar compositions to those extracted from whole cells but had a very low degree of unsaturation [48].

Comparison of our dataset with the transcriptomic data reported by Mock et al. (2008) [10] and Nemoto et al. (2020) [44], and proteomic data reported by Frigeri et al. (2005) [49] and Skeffington et al. (2022) [29], showed good agreement with that these genes encoding proteins for methyltransferases and SITs would play important roles in diatom biosilicification. In contrast, other significantly enriched biological processes such as transcription factors (especially heat shock factors), cGMP-mediated signaling pathways, cytoskeleton associated, sugar and fatty acid metabolism were not previously described at the transcriptional level in other diatoms. In addition, half of DEGs encoding proteins with predict or unknown functions were newly discovered in the present study. Developing genome-editing tools to define the functions of these candidate genes in *N. closterium* f. minutissima would provide sufficient evidence to support their contributions to silicon deposition in the future.

## Conclusion

In conclusion, the present study obtained the draft genome of *N. closterium* f. minutissima, also termed *P. tricornutum*_PRJNA943072, and revealed that it was most closely related to *P. tricornutum* among the nine diatom species. However, further analysis revealed that these genes exhibited different features of gene expansion and contraction. Subsequently, transcriptome analyses were performed, and numerous DEGs in response to silicon were identified in *P. tricornutum*_PRJNA943072, these genes are involved in various biological processes. Overall, SITs, the second messenger cGMP-mediated signalling pathways, and transcription factors such as heat shock factors seem to play important roles in silicon transport, signal transduction and transcriptional activation of genes. Cytoskeleton-associated proteins, polyamines, glycoproteins and saturated fatty acids were likely to constitute frustules during diatom biosilicification. In addition, genes encoding methyltransferases and ankyrin repeat domain proteins are worthy of further study.

## Methods

### Algal strain and culture conditions

*N. closterium* f. minutissima was obtained from the Center for Collections of Marine Algae of Xiamen University. For genome sequencing, the algal cells were cultured in 1/2f medium and maintained at 23 °C under continuous illumination at a light density of 150 µmol·m^− 2^·s^− 1^. The cells were collected on a filter membrane (0.8 µM of 50 mm; Xinya, China) with a diaphragm vacuum pump (Jinteng GM-2, Tianjin, China) when the concentration reached 2 × 10^6^ cells/mL. The samples were immediately frozen in liquid nitrogen and stored at -80 °C. For transcriptome analysis, the cells were cultured in 1/2f medium to reach a growth plateau and then collected by centrifugation (3,000 × g). After being washed twice with 1/2f medium deficient in silicate (1/2f-Si medium), the cells were inoculated in 1/2f –Si medium for at least 24 h to obtain a synchronized starter culture. Then, the cultures were collected again by centrifugation and resuspended in normal 1/2f medium containing silicate. Samples were collected and frozen after reculturing with silicon for 0, 6 and 12 h for RNA-seq.

### Genome sequencing and assembly

Genome sequencing was performed at Nextomics Biosciences Co., Ltd. (Wuhan, China). The genomic DNA was extracted with a QIAGEN® Genomic Kit (QIAGEN, Germany) according to the manufacturer’s instructions. The DNA concentration was measured by a Qubit® 3.0 fluorometer (Invitrogen, USA), and the integrity was checked by agarose gel electrophoresis. A total of 2 µg of long DNA fragments were extracted from agarose gels using the BluePippin system (Sage Science, USA). Next, the ends of the DNA fragments were repaired, and A-ligation reaction were conducted with an NEBNext Ultra II End Repair/dA-tailing Kit. The adapter in the LSK109 kit was used for further ligation and a Qubit® 3.0 fluorometer (Invitrogen, USA) was used to quantify the size of the library fragments. Sequencing was then a performed on a PromethION sequencer (Oxford Nanopore Technologies, UK).

After quality control of the raw reads, the pass reads were subject to de novo genome assembly via an OLC (overlap layout-consensus)/ string graph method of NextDenovo. The original subreads were first self-corrected using the NextCorrect module to obtain consistent sequences (CNS reads), and the preliminary genome was subsequently assembled based on the correlation of the CNSs captured by the NextGraph module. To improve the accuracy of the assembly, the contigs were refined with Racon using ONT long reads and Nextpolish using Illumina short reads with default parameters. To evaluate the accuracy of the assembly, all the Illumina paired-end reads were mapped to the assembled genome using Burrows-Wheeler Aligner (BWA), and the mapping rate and genome coverage of the sequencing reads were assessed using SAMtools v0.1.1855. In addition, the base accuracy of the assembly was calculated with BCFtools. The coverage of expressed genes in the assembly was examined by aligning all the RNA-seq reads against the assembly using HISAT with default parameters. To avoid including mitochondrial sequences in the assembly, the draft genome assembly was submitted to the NT library, after which the aligned sequences were eliminated.

### Gene prediction and annotation

The simple repeat sequences (SSRs) and tandem repeat elements were recognized by the software GMATA v2.2 and Tandem Repeats Finder (TRF), respectively. Transposable elements (TE) were identified by using a combination of ab initio and homology-based methods. Briefly, an ab initio repeat library was first predicted using MITE-Hunter and RepeatModeller v1.0.11 with default parameters, after which the obtained library was aligned to the TEclass Repbase (http://www.girinst.org/repbase) to classify the type of each repeat family. To further identify repeats throughout the genome, RepeatMasker v1.331 was applied to search for known and novel TEs by mapping sequences against the de novo repeat library and Repbase TE library. Redundant TEs belonging to the same repeated class were deleted.

For gene prediction, three independent approaches including homology search, reference guided transcriptome assembly and ab initio prediction were used in a repeat-masked genome. A homology search was performed with GeMoMa v1.6.1 software for homologous proteins from related species, including *T. pseudonana*, *Nitzschia multistriata*, *F. cylindrus*, *T. oceanica*, *Fistulifera solaris* and *P. tricornutum*. Reference guided transcriptome assembly was carried out by using STAR v2.7.3a, Stringtie v1.3.4d and PASA v2.3.3 software with default parameters. The software Augustus v3.3.1 were applied for ab initio gene prediction with a training set produced by the software PASA v3.3.1 and GeneMark-ST. Finally, EVidenceModeller (EVM) v1.1.1 was used to produce an integrated gene set in which gene with TEs were removed using the TransposonPSI package (http://transposonpsi.sourceforge.net/), and the miscoded genes were further filtered. Untranslated regions (UTRs) and alternative splicing regions were determined using PASA based on RNA-seq assemblies. We retained the longest transcripts for each locus, and regions outside of the ORFs were designated UTRs.

Gene functions were assigned by aligning the protein sequences against public databases, including SwissProt, NR, KEGG, KOG and GO. The putative domains and GO terms were identified using InterProScan V 5.32 with default parameters, and the BLASTp program was used for the other four databased at expected values (E) of < 10 − 5.

To obtain the ncRNA (noncoding RNA), two strategies were used: searching against the database and prediction with the model. Transfer RNAs (tRNAs) were predicted using tRNAscan-SE with eukaryotic parameters. MicroRNA, rRNA, small nuclear RNA, and small nucleolar RNA were detected using Infernal cmscan to search the Rfam database. The rRNAs and their subunits were predicted using RNAmmer.

### Phylogenetic analysis and gene family evolution

The protein sequences of 8 diatom species including *C. tenuissimus*, *F. cylindrus*, *P. tricornutum*, *Pseudo n. multistriata*, *S. robusta*, *T. oceanica*, *T. pseudonana* and *F. crotonensis* were downloaded from the NCBI database and used to identify single-copy orthologue sequences by using OrthoFinder v2.3.14 [50] with the protein sequences of *N. closterium* f. minutissima together. A phylogenetic analysis was performed by using the software PhyloSuite v1.2.3 [51, 52] as follows: the single-copy sequences were aligned with MAFFT v7.505 [53] using an auto strategy and normal alignment mode and concatenated into a supermatrix for each species. ModelFinder v2.2.0 [54] was subsequently used to select the best-fit partition model (Edge-linked) using the Bayesian information criterion (BIC), and the phylogenetic tree was ultimately constructed by using IQ-TREE v2.2.0 [55] under edge-linked partition model [56] for 5000 ultrafast bootstraps. The species divergence time was estimated using MCMCTREE in PAML v4.9j [57]. The *C. tenuissimus*–*T. pseudonana* divergence (~ 162–187 Mya) and *F. crotonensis*–*F. cylindrus* (~ 93–104 Mya) were obtained from TIMETREE 5 (http://timetree.org/) and used as fossil calibration points. Gene family expansion and contraction were inferred using CAFE v5.0 [58]. The phylogenetic tree was displayed and annotated using TVBOT (https://www.chiplot.online/tvbot.html) [59]. Diagrams of Venn and UpSet plots were drawn by using VennMaster [60] and TBtool [61], respectively.

### Cell synchronization

Cell synchronization was performed as described previously with some modifications [49]. Briefly, the algal cells were grown in 1/2f medium to a concentration of about 2 × 10^6^/mL, and subsequently transferred into silicate-free 1/2f medium under sterile conditions. After 24 h, silicate was added back to the culture at a final concentration of 106 µM. Then a small aliquot of cells was stained with 2 µg/mL rhodamine 123 (final concentration) and imaged by fluorescence microscopy every hour for 12 h.

### RNA-seq and transcriptome analysis

After synchronization with silicate-free culture medium, the algal cells were grown in 1/2 medium for 0, 6–12 h and subsequently collected. Total RNA was extracted by using an RNAprep Pure Plant Plus Kit (Tiangen, China) according to the manufacturer’s instructions. The RNA-seq was performed on an Illumina NovaSeq platform at Novogene (Beijing, China; https://cn.novogene.com/). The experiment was repeated three times. The raw sequences were quality-filtered and mapped to the *N. closterium* f. minutissima genome using HISAT2 v2.0.5. Differential expression analysis of two conditions/groups (two biological replicates per condition) was performed using the DESeq2 R package (1.20.0). A corrected P-value of 0.05 and an absolute foldchange of 2 were set as the thresholds for significant differential expression. After differential gene expression analysis, the differentially expressed genes were subjected to Gene Ontology (GO) enrichment analysis (http://www.geneontology.org/).

### Electronic supplementary material

Below is the link to the electronic supplementary material.

Supplementary Material 1

Supplementary Material 2

Supplementary Material 3

## Data Availability

This whole genome sequence has been deposited at DDBJ/ENA/GenBank under the accession JARGZD000000000. The version described in this paper is version JARGZD010000000, and the BioProject is PRJNA943072. The raw data are available in the NCBI Sequence Read Archive (SRA) database (https://www.ncbi.nlm.nih.gov/sra) under accession no. SRR23852742 and SRR23852743. The RNA-seq data have been deposited in the NCBI database under BioProject PRJNA943172, and the raw data are available in the NCBI Sequence Read Archive (SRA) database under accession no. SRR23849345-SRR23849353.

## Abbreviations

DEGs: Significantly differentially expressed genes
GO: Gene Ontology
HSF: Heat shock factor
KEGG: Kyoto Encyclopedia of Genes and Genomes
RNA-seq: RNA sequencing
SDV: Silica deposition vesicle
SIT: Silicon transporter
