# Supplementary material for: The draft genome of Nitzschia closterium f. minutissima and transcriptome analysis reveals novel insights into diatom biosilicification
Source: BMC Genomics. 2024 Jun 5;25:560. doi: 10.1186/s12864-024-10479-9 (PMC11151724; doi:10.1186/s12864-024-10479-9)
Supplement: Supplementary file 1 — Supplementary Material 1 [file 12864_2024_10479_MOESM1_ESM.docx]

| Class | Order | Super family | Number of elements | Length of sequence (bp) | Percentage of sequence (%) |
| --- | --- | --- | --- | --- | --- |
| Class I |  |  |  |  |  |
|  | LTR |  | 3,464 | 4,204,996 | 14.36 |
|  |  | Copia | 2,500 | 3,767,476 | 12.87 |
|  |  | Unknown | 790 | 413,126 | 1.41 |
|  |  | Other | 174 | 24,394 | 0.08 |
|  | LINE |  | 437 | 151,214 | 0.52 |
|  |  | Unknown | 372 | 138,456 | 0.47 |
|  |  | Other | 65 | 12,758 | 0.04 |
|  | SINE |  | 6 | 896 | 0 |
|  |  | Other | 6 | 896 | 0 |
| Class II |  |  |  |  |  |
|  | DNA |  | 1,354 | 684,073 | 2.34 |
|  |  | Unknown | 822 | 408,520 | 1.4 |
|  |  | hAT-Charlie | 149 | 47,946 | 0.16 |
|  |  | MULE-MuDR | 87 | 49,674 | 0.17 |
|  |  | PiggyBac | 124 | 131,353 | 0.45 |
|  |  | Other | 172 | 46,580 | 0.16 |
|  | RC |  | 31 | 2,843 | 0.01 |
|  |  | Other | 31 | 2,843 | 0.01 |
| Tandem Repeats |  |  | 2,184 | 124,581 | 0.43 |
|  | tandem_repeat |  | 805 | 106,063 | 0.36 |
|  | SSR |  | 1,379 | 18,518 | 0.06 |
| Simple repeats |  |  | 102 | 12,368 | 0.04 |
| Unknown |  |  | 514 | 192,673 | 0.66 |
| Other |  |  | 29 | 1,637 | 0.01 |
| Low complexity |  |  | 1 | 203 | 0 |
| Total Repeats |  |  | 8,122 | 5,375,484 | 18.36 |

Table S1 Statistics of repetitive elements of the genome assembly

Table S2 Taxa classification of diatom species used for the construction of phylogenetic tree in this study.

| species | Phylum | Subphylum | Class | Subclass | Order | Family | Genus |
| --- | --- | --- | --- | --- | --- | --- | --- |
| *Phaeodactylum tricornutum* | Heterokontophyta |  | Bacillariophyceae  incertae sedis |  | Bacillariophyceae  ordo incertae sedis | Phaeodactylaceae | Phaeodactylum |
| *Seminavis robusta* | Heterokontophyta | Bacillariophytina | Bacillariophyceae | Bacillariophycidae | Naviculales | Naviculaceae | Seminavis |
| *Fragilariopsis cylindrus* | Heterokontophyta | Bacillariophytina | Bacillariophyceae | Bacillariophycidae | Bacillariales | Bacillariaceae | Fragilariopsis |
| *Pseudo nitzschia multistriata* | Heterokontophyta | Bacillariophytina | Bacillariophyceae | Bacillariophycidae | Bacillariales | Bacillariaceae | Pseudo-nitzschia |
| *Fragilaria crotonensis* | Heterokontophyta | Bacillariophytina | Bacillariophyceae | Fragilariophycidae | Fragilariales | Fragilariaceae | Fragilaria |
| *Thalassiosira oceanica* | Heterokontophyta | Bacillariophytina | Mediophyceae | Thalassiosirophycidae | Thalassiosirales | Thalassiosiraceae | Thalassiosira |
| *Thalassiosira pseudonana* | Heterokontophyta | Bacillariophytina | Mediophyceae | Thalassiosirophycidae | Thalassiosirales | Thalassiosiraceae | Thalassiosira |
| *Chaetoceros tenuissimus* | Heterokontophyta | Bacillariophytina | Mediophyceae | Chaetocerotophycidae | Chaetocerotales | Chaetocerotaceae | Chaetoceros |
